# Direct Medical Costs Associated with Multiple Sclerosis in Saudi Arabia: A Retrospective Single-Center Cost of Illness Analysis

**DOI:** 10.3390/healthcare13243229

**Published:** 2025-12-10

**Authors:** Ahmed Alghamdi, Hamoud Almutairi, Ghada Alnasser, Shoroq Alsoina, Najwa Aljaber

**Affiliations:** Department of Clinical Pharmacy, College of Pharmacy, King Saud University, Riyadh 11451, Saudi Arabia; halmotairi@ksu.edu.sa (H.A.); ghada.4869@gmail.com (G.A.); shoroqalsoina@gmail.com (S.A.); 444203117@student.ksu.edu.sa (N.A.)

**Keywords:** multiple sclerosis, direct medical cost, cost of illness, prevalence-based, public healthcare payer perspective, Saudi Arabia

## Abstract

**Background/Objectives**: Globally, multiple sclerosis (MS) management is associated with substantial economic burden, but its impact in Saudi Arabia has not been fully quantified. The aim of this study was to estimate the direct medical costs of MS in Saudi Arabia and identify its main cost drivers from a public healthcare payer perspective. **Methods**: A retrospective, prevalence-based, single-center cost-of-illness analysis was conducted between 2019 and 2020. A bottom-up micro-costing approach was used to estimate the annual direct medical costs. Descriptive and inferential statistics were used. **Results**: A total of 193 patients with MS were included (mean age, 33 years; 62% female; 66% with relapsing-remitting MS). Overall, 48% of patients were at a mild disease stage, and 57% had a disease duration of less than five years. The total cost incurred during the study period was $4,157,436. The annual direct medical cost per patient is $21,541 ± 1475. Costs were significantly associated with EDSS score and frequent relapses, resulting in an increase of $729 and $1349, respectively. Additionally, disease-modifying therapies were identified as a major cost driver (74%). **Conclusions**: MS poses a significant financial burden on the Saudi healthcare system. The results of this study can inform policy development and guide resource allocation in planning healthcare services for patients with MS.

## 1. Introduction

Multiple sclerosis (MS) is a chronic autoimmune disease that affects the central nervous system, leading to significant neurological deficits and severe disability [1,2,3]. The prevalence of MS has increased markedly over the past decade in both developed and developing countries, with a global estimate of 2.8 million affected individuals [4,5]. Although MS can occur at any age, its prevalence is highest among individuals aged 20–50 years [6]. In addition, MS affects females more frequently than males, with approximately 60% of reported cases occurring in females [7].

Although the countries of the Gulf Cooperation Council (GCC) have historically been considered a low-risk zone, the prevalence of MS in Saudi Arabia and other GCC countries has risen over the years [8,9]. In Saudi Arabia, the estimated prevalence of MS is 40.40 per 100,000 of the general population, increasing to 61.95 per 100,000 among Saudi nationals [10]. The mean age at diagnosis is 33.43 years, and 92.6% of patients with MS have a relapsing form of the disease. Furthermore, the female-to-male ratio is approximately 2:1, and nearly 70% of patients with MS are between 21 and 40 years old [11].

MS is a progressive condition and one of the leading causes of disability, exerting a substantial negative impact on patients’ physical, mental, and social well-being, as well as their quality of life [12]. Patients with MS often experience relapses and exacerbations of neurological symptoms, resulting in frequent hospitalizations and increased use of healthcare services [13,14]. Owing to its complexity and higher prevalence among younger, working-age individuals, MS also carries significant societal consequences, including loss of productivity, absenteeism, and early retirement [15]. Currently, no cure for MS exists, but available treatment options, including disease-modifying therapies (DMTs), can effectively reduce relapse rates and improve long-term prognosis and quality of life. Additional interventions such as symptomatic management, lifestyle modification, rehabilitation, and psychological support may also contribute to modest improvements in the quality of life and reductions in productivity loss [16].

Despite advances in treatment, MS continues to impose a considerable economic burden on patients, families, healthcare systems, and society. This burden arises from its increasing prevalence, clinical complexity, high resource utilization, demand for caregiving, and associated productivity losses [17,18]. The economic impact of MS has been estimated in several countries. For example, in the United States, the total annual cost of MS management has reached $85.4 billion, with almost 74% attributed to direct medical costs [19]. In European countries such as Italy, France, and the United Kingdom (UK), the estimated total annual costs were €4.8 billion, €2.7 billion, and £1.4 billion, respectively, in which direct medical costs were the major driver of the overall cost [20,21,22].

At the patient level, the average annual cost of MS varies across countries. The average cost per patient in the United States, Australia, and other high-income European countries has been estimated at $88,487, $73,457, and €40,303, respectively [19,23,24]. In contrast, the average annual cost per MS patient in low–middle income countries are lower, ranging from $463 to $58,616 [25]. Although differences in methodological approaches have led to variations in cost estimates between countries, most studies report that costs increase with disease progression and greater disability. Direct medical costs, particularly those related to medications, such as DMTs and hospitalizations, are the main cost drivers in the early stages of MS. In contrast, indirect costs, including those associated with productivity loss, become more significant in advanced stages of the disease [25,26].

Despite the notable increase in MS prevalence, evidence regarding its financial impact on Saudi healthcare system remains limited. A detailed estimation of MS-related healthcare costs in Saudi Arabia would provide valuable insights about the financial impact of the disease. Such information is increasingly important given the recent transformation of the Saudi healthcare system toward a value-based care model and the growing privatization of healthcare services as part of the country’s 2030 vision, where financial considerations play a critical role in decision-making [27]. Therefore, this study aims to estimate the annual cost of MS management from a public healthcare payer perspective, over a one-year period of patients diagnosed with MS, and to identify the key cost drivers. In addition, the study aimed to explore the expected impact of disease characteristics including (MS phenotype, disease progression, and relapse rate) on healthcare costs associated with MS. The outcomes of this study could help healthcare policymakers in accurately estimating MS-related costs and in developing policies to optimize resource allocation and control healthcare spending.

## 2. Materials and Methods

### 2.1. Study Design, Population, and Data Collection

This was a retrospective, prevalence-based cost-of-illness (COI) study conducted at King Saud University Medical City (KSUMC) in Riyadh, Saudi Arabia. This study was carried out from a public healthcare payer perspective to capture the direct medical costs. To estimate the direct medical costs, a COI model was developed based on the cost of individual service units. MS patients were identified from the electronic medical records (EMRs) using International Classification of Diseases 10th Revision (ICD-10) coding system. The inclusion criteria were the following: conformed diagnosed of MS based on the 2017 McDonald criteria [28], aged 18 years or older, and treatment with DMT for at least 12 months. The time horizon for this study was one year from September 2019 to September 2020. Patients were excluded if they did not fit the inclusion criteria, classified as clinically isolated syndrome or radiologically isolated syndrome, or had specific comorbidities including (cancer, cardiovascular diseases, chronic kidney disease). In addition, patients’ records with incomplete or with missing data were excluded.

Patient demographics and disease characteristics were collected, including age, sex, marital status, smoking status, disease duration, number of MS relapses, phenotype, and disease stage. The MS phenotype was determined based on disease activity, as reflected by relapses and progression. Accordingly, patients were classified as having either relapsing-remitting MS (RRMS) or non-relapsing MS, which included secondary progressive MS (SPMS) and primary progressive MS (PPMS) [29]. Disease stage was determined using the Expanded Disability Status Scale (EDSS) reported in the EMRs. Patients were categorized as having mild MS (EDSS score 0–3), moderate MS (EDSS score 3.5–5.5), or severe MS (EDSS score 6–9) [30].

### 2.2. Direct Cost Estimation

The cost of healthcare resources directly associated with MS treatment were estimated using a bottom-up, micro-costing method. First, the healthcare resources and interventions used by each patient during the study period were identified and quantified. The unit costs of these interventions were then determined, and the total direct cost per patient was estimated by multiplying the unit cost by the quantity consumed. Healthcare service cost data, including hospitalization, procedures, medications, diagnostic tests, laboratory tests, neurology outpatient visits, and emergency visits, were obtained from the KSUMC Business Center. To avoid overestimation, overhead costs were excluded from the analysis, since it is not tied to a specific patient or service. The total direct cost per patient was calculated on an annual basis, with all cost calculations adjusted for inflation to the year 2025. All cost calculations were expressed in United States dollars (USD), with an exchange rate of 1 USD = 3.75 Saudi Riyal.

### 2.3. Statistical Analysis

Descriptive statistics were generated, including means, standard deviations (SD), and frequencies. Inferential statistics, including t-tests and analysis of variance, were used to compare MS-related costs across different disease characteristics including MS phenotype, stage, and relapse rate. To identify predictors of healthcare cost associated with MS, a regression analysis was performed using a generalized linear model, which is commonly used in COI analysis to handle skewness and heteroskedasticity in cost data. The model included factors such as age, gender, disease duration, relapse rate, and stage (based on the EDSS score). Statistical significance was set at *p* < 0.05. All analyses were performed using the Statistical Package for the Social Sciences (SPSS) version 21 (SPSS Inc., Chicago, IL, USA).

## 3. Results

A total of 193 patients with MS were included in the study. Patient demographics and disease characteristics are summarized in Table 1. Two-thirds of the study population were female (62%), and the mean age was 33 ± 9 years, with more than half of the patients (53%) aged under 35 years. Most participants were employed (65%) and non-smokers (76%). In addition, two-thirds of the study population were classified as RRMS (66%), and more than half (57%) had a disease duration of less than five years. Furthermore, 48% were in the mild stage of MS, and 52% had two or fewer relapses during the study period.

### Direct Costs

The total direct medical costs associated with MS management during the study period were estimated at $4,157,436. The average annual direct medical cost per patient was $21,541 ± 1475. The total and average direct medical costs varied according to patient demographics and disease characteristics (Table 2). Overall, patients with the following characteristics accounted for the largest proportion of total medical costs: female gender (67%), age ≤ 35 years (53%), RRMS (72%), mild MS stage (45%), and more than two relapses during the study period (53%).

The average direct medical cost was higher among female patients than male patients ($23,135 and $18,921, respectively). Patients aged 35 years or younger had a slightly higher average direct medical cost than those older than 35 years ($22,465 vs. $20,588). The average direct medical cost associated with the RRMS phenotype was $23,337, compared with $18,004 for non-RRMS (PRMS and SPMS). Average costs also varied significantly by MS stage, ranging from $19,949 in the mild stage to $24,057 in the severe stage (*p* = 0.004). Additionally, patients who experienced more than two relapses incurred significantly higher average direct medical costs than those with two or fewer relapses ($24,117 vs. $19,195; *p* = 0.03).

**Figure 1:**
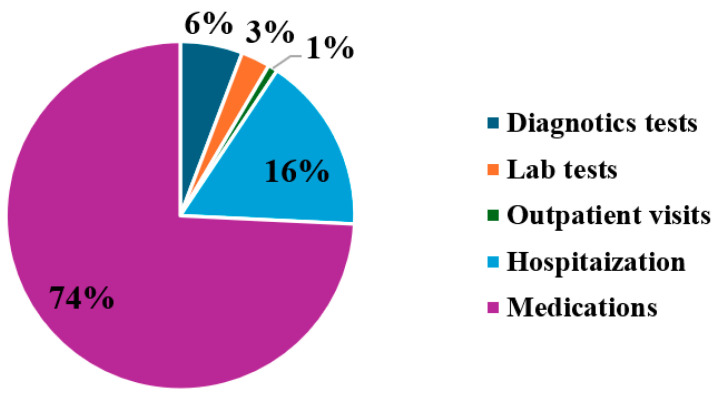
Major drivers of direct healthcare costs associated with MS management.

Regression analysis revealed that age, gender, and diseases duration were not significantly associated with direct medical cost. Conversely, direct medical costs were significantly associated with MS EDSS scores, in which each additional point was associated with an increase of $729, 95% CI [$590, $868] (*p* = 0.002). Moreover, direct medical costs were significantly associated with the number of MS relapses, in which each relapse event associated with an increase of $1349, 95% CI [$1025, $1673] (*p* = 0.005). The main drivers of direct medical costs across the study population are shown in Figure 1. Hospitalization costs, which included cost related to in-patient services during hospital stays resulting from MS exacerbations, accounted for 16% and average annual cost of $3355, while diagnostic tests, outpatient visits, and laboratory tests accounted for 6%, 3%, and 1%, and average annual costs of ($1245, $581, $191), respectively. Overall, the study found that medication costs, particularly those related to DMTs, were the primary contributors to MS management expenditures (74%) and with an average cost of $15,992. Among these, approximately 68% of medication costs were attributed to interferon beta-1a, followed by fingolimod (13%), natalizumab (8%), teriflunomide (6%), and interferon beta-1b (2%) (Table 3).

## 4. Discussion

MS is a progressive neurological disorder that causes lifelong disabilities and has substantial physical, mental, and social impacts. Globally, MS imposes a heavy economic burden on healthcare systems, payers, and societies, leading to significant government expenditures and loss of income for patients and their caregivers [31,32]. Estimating the financial impact of MS is therefore essential for understanding healthcare resource utilization and total healthcare spending on this disease. To the best of our knowledge, this is the first study to quantify the healthcare costs associated with MS in Saudi Arabia and it provides insights into the costs associated with MS management across different patient groups and disease characteristics, which will help decision-makers to prioritize healthcare policies, implement interventions, and allocate available health resources more efficiently.

The findings indicate that MS imposes a considerable financial burden in Saudi Arabia. The total cost associated with managing 193 patients with MS was estimated at $4,157,436, Moreover, the estimated average annual direct medical cost per patient is $21,541, higher than in neighboring countries such as Lebanon, Jordan, and Kuwait ($11,252, $17,185, and $17,296, respectively) [33,34,35]. These variations in cost estimates reported from different countries may be due to the methodology used for estimating costs, MS treatment protocols, access to healthcare resources, and prices of healthcare technologies used in MS management. This variation underscores the importance of using local data to estimate the actual costs of MS. Relying on data from other countries may misrepresent actual costs, which in turn affect decisions related to healthcare spending and resource allocation.

The major contributor to overall direct medical costs was medication, representing 74% of the total. DMTs, particularly Interferon Beta-1a, Fingolimod, Natalizumab, and Teriflunomide, accounted for 95% of medication costs, reflecting the high prices of DMTs in Saudi Arabia. These results align with previous studies in other countries, which similarly identified DMT costs as the primary driver of direct medical expenses in MS management [36,37]. However, our findings contrast with reports from Germany and the UK, where DMTs account for a smaller proportion of total direct medical costs, due to lower DMT prices in those countries [38,39].

DMT utilization was higher in the early stages of MS (mild-to-moderate) than in severe stages. Despite a reduction in DMT use with increasing disease severity, their costs remain the leading contributor to direct medical expenses, surpassing other drivers such as hospitalization, diagnostic tests, and healthcare provider visits. Although DMTs are expensive and pose affordability challenges for patients and payers, they offer significant clinical benefits, including reductions in relapse frequency and disability progression. Early treatment with DMTs can also delay MS progression, thereby reducing the long-term use of healthcare resources [40]. These findings highlight the importance of strategies that balance access, clinical value, and affordability of DMTs for patients with MS in Saudi Arabia.

Direct medical costs also varied according to demographics and disease characteristics. Female patients and those aged 35 years or younger tended to utilize more medical resources, resulting in higher average direct medical costs than male patients and those over 35 years. These differences reflect higher MS prevalence among women and younger patients, as well as the tendency for younger patients to receive more aggressive treatment regimens. However, these variations are not statistically significant and are consistent with previous reports [40,41].

When analyzing MS costs by phenotype, this study found that patients with RRMS comprised 66% of the total study population and accounted for 72% of total direct medical costs. Higher relapse activity is significantly associated with increased direct medical costs, primarily because more frequent relapses require greater use of symptomatic treatments, healthcare provider visits, emergency room visits, and hospitalizations. Similar patterns have been reported in studies from Canda, the United States, the Netherlands, and Turkey, which demonstrated higher costs associated with RRMS and severe relapses. Additionally, a US study found that patients with high relapse activity utilized more healthcare resources than those with lower relapse activity [42].

Direct costs of MS management also increase significantly as the disease progresses to advanced stages, reflecting higher levels of disability. Average direct medical costs range from $19,949 for patients in the mild stage to $24,057 for those in the severe stage, with costs rising as EDSS scores increase. These findings indicate that in Saudi Arabia, higher MS disability is directly associated with increased direct medical costs, due to greater treatment requirements and higher consumption of healthcare resources by patients with advanced disability. These results, aligned with previous studies, show that direct medical costs escalate with disease progression, increasing EDSS scores, and greater disability [43,44,45,46].

Overall, this study highlights the financial burden of MS in Saudi Arabia, emphasizing its relevance for healthcare decision-makers. Limited healthcare resources and the impact of MS on patient productivity create a pressing need for strategies that reduce costs while sustaining healthcare service delivery. One approach is to lower the costs of DMTs by improving patient access to these medications at more affordable prices, considering typically constrained healthcare budgets.

Potential strategies include switching from expensive injectable DMTs to oral DMTs for patients with highly active MS, which could improve outcomes and reduce costs [47]. Another approach is to promote the approval and uptake of biosimilars and generic DMTs, which can provide significant cost savings, reduce the prices of original products, and improve patient access to essential treatments [48].

Furthermore, evaluating the added value and affordability of DMTs, which can vary by country, is essential before including them in hospital formularies or national treatment protocols. The application of pharmacoeconomic principles, such as cost-effectiveness and budget impact analyses, would be particularly valuable for assessing the value and affordability of these expensive medications. Currently, there are limited number of studies that evaluated the added value of DMT in MS management in Saudi Arbia, and the results of these studies varied, relying mainly on published cost data, clinical trials data, or efficacy data from other countries [49,50]. Conducting such analyses within a local Saudi context would enhance the accuracy and reliability and could help optimizing the use of limited healthcare resources. These findings could inform policies to improve payment models and support a value-based healthcare approach that links patient-reported outcomes to reimbursement. Such strategies are critical for sustaining the healthcare system while maintaining or improving quality of care and controlling costs.

Increasing public and healthcare awareness and improving early detection of MS are also essential policy initiatives to reduce costs associated with managing advanced disease. Evidence indicates that early diagnosis and treatment can lower overall healthcare expenditures by reducing long-term complications and slowing disease progression and disability [48].

### Limitations

This study has several limitations that should be noted. First, this study was conducted at a single large tertiary hospital and did not include patients from other major institutions or the private sector. In addition, being conducted at a single center adds concerns about selection bias and accuracy of data retrieved from the EMR. Moreover, most patents were females, had RRMS phenotype, and classified as mild-to-moderate disease stage, which might limit the generalizability of the findings. Therefore, total population cost estimates should be interpreted with caution. Second, although several confounders were included in the regression analysis, the study did not account for certain variables that are linked to MS, including obesity and vitamin D deficiency. Third, the estimation of costs focused entirely on direct medical costs and did not account for non-medical costs, such as informal care, housing, transportation, or other services. Moreover, the study did not include the estimation of indirect costs, which has been proven to have significant impact on the economic burden of MS due to productivity loss resulting from disability and progression [26]. Future research should consider nationally representative MS samples and include additional cost components, such as direct non-medical and indirect costs, which could substantially affect the overall economic burden, especially for patients with advanced disease.

## 5. Conclusions

This study provides important insight into the financial impact of MS in Saudi Arabia. Overall, MS poses a significant financial burden, primarily driven by the excessive costs of DMTs. The findings offer valuable information for health policy decision-makers regarding healthcare resource utilization and expenditures associated with MS. This evidence can support the prioritization of healthcare policies, the implementation of effective interventions, efficient allocation of resources, and management of the disease. The findings highlight the importance of public awareness, early detection of MS, and the use of generic or biosimilar forms of DMT as tools to help lower costs and increase savings. Future research is required to comprehensively evaluate the full economic impact of MS by adapting social perspective and national representative data. In addition, evaluating the added value and the potential affordability of new and expensive DMT could help improve patients’ outcomes while controlling healthcare costs.

## Figures and Tables

**Table 1:** Study population demographics and disease characteristics.

Variable	Count (n = 193)	Frequency (%)
**Gender**		
Male	73	38%
Female	120	62%
**Age (mean, SD 33 ± 9)**		
≤35	102	53%
>35	91	47%
**Marital status**		
Single	97	50%
Married	85	44%
Other	11	6%
**Employment Status**		
Employed	126	65%
Unemployed	67	35%
**Smoking**		
No	147	76%
Yes	46	24%
**Disease duration (mean, SD 6.4 ± 3)**		
≤5 years	110	57%
>5 years	83	43%
**MS Phenotype**		
RRMS	128	66%
Non-RRMS	65	34%
**MS stage based on EDSS (mean, SD 2.9 ± 1)**		
Mild	94	48%
Moderate	71	37%
Severe	28	15%
**Relapses**		
≤2	101	52%
>2	92	48%

Abbreviations: EDSS, Expanded Disability Status Scale; MS, multiple sclerosis; RRMS, relapsing-remitting multiple sclerosis; Non-RRMS includes both primary progressive multiple sclerosis (PPMS) and secondary progressive (SPMS); SD: standard deviation.

**Table 2:** Direct medical costs associated with MS in the study population.

Variable	Toal Cost	Mean	SD	% of Total Costs
**Gender**				
Male	$1,381,227	$18,921	$1378	33%
Female	$2,776,209	$23,135	$1459	67%
**Age**				
≤35	$2,201,531	$22,465	$1441	53%
>35	$1,955,905	$20,588	$1382	47%
**MS Phenotype**				
RRMS	$2,987,163	$23,337	$1403	72%
Non-RRMS	$1,170,274	$18,004	$1374	28%
**MS Stage**				
Mild	$1,875,164	$19,949	$1397	45%
Moderate	$1,608,685	$22,658	$1587	39%
Severe	$673,587	$24,057	$2098	16%
**Relapses**				
≤2	$1,938,695	$19,195	$1485	47%
>2	$2,218,741	$24,117	$1933	53%

Abbreviations: MS, multiple sclerosis; RRMS, relapsing-remitting multiple sclerosis; non-RRMS, primary progressive multiple sclerosis (PPMS) and secondary progressive (SPMS); SD, standard deviation.

**Table 3:** Utilization and costs of MS treatment medication during study period.

Drug	Utilization	Total Cost	% of Total Drug Costs
Interferon Beta-1a	28%	$2,103,944	68%
Fingolimod	9%	$403,545	13%
Natalizumab	8%	$232,497	8%
Teriflunomide	6%	$195,130	6%
Interferon Beta-1b	2%	$59,138	2%
Rituximab	6%	$44,907	1%
Ocrelizumab	1%	$42,565	1%
Other Drugs	40%	$15,790	1%

Note: other drugs include drugs used as supplements, immuno-suppressants, or to help control MS symptoms and relapses including Mycophenolate mofetil, Methotrexate, Cholecalciferol, Hydroxychloroquine, Carbamazepine, Topiramate, Azathioprine.

## Data Availability

The original contributions presented in this study are included in the article. Further inquiries can be directed to the corresponding author.

## Abbreviations

The following abbreviations are used in this manuscript:

MS	multiple sclerosis
GCC	Gulf Cooperation Council
DMTs	disease-modifying therapies
COI	cost-of-illness
KSUMC	King Saud University Medical City
EMRs	electronic medical records
RRMS	relapsing-remitting MS
SPMS	secondary progressive MS
PPMS	primary progressive MS
EDSS	Expanded Disability Status Scale
